# The End-Results of Operations on the Stomach and Duodenum

**Published:** 1911-09

**Authors:** A. Rendle Short

**Affiliations:** Hon. Surgical Registrar, Bristol Royal Infirmary; Senior Demonstrator of Physiology, University of Bristol


### THE END-RESULTS OF OPERATIONS ON THE STOMACH AND DUODENUM.

BY

A. Rendle Short, B.Sc., M.D., B.S., F.R.C.S.,
*Hon. Surgical Registrar, Bristol Royal Infirmary ;*
*Senior Demonstrator of Physiology, University of Bristol.*

The conclusions embodied in this article are drawn from a study of the records of all the stomach cases treated in the surgical wards of the Bristol Royal Infirmary during the years 1902–10 inclusive.

The number of patients under consideration has been 165, out of a total of 19,412 treated in the surgical wards during the same period.

They may be classified as follows, according to the result of investigation :—

| | |
|---|---|
| Subsequent information obtainable .. | 75 |
| Lost sight of .. .. .. .. .. .. .. | 30 |
| Died .. .. .. .. .. .. .. .. .. .. | 60 |
| Total .. .. .. .. | 165 |

The cases followed have all been communicated with not sooner than four months after the operation ; all except two have been traced for more than six months, and the great majority give a history extending over several years since they left the Infirmary. I hope, therefore, that this may be accepted as a disinterested report on an adequate number of cases, followed sufficiently long after the operation to give reliable indications. The after-histories were obtained in most cases by correspondence, but a few have been seen and examined by me personally.

The morbid conditions represented are as follows :—

| | |
|---|---|
| 61 cases of perforated gastric or duodenal ulcer, with after-history .. .. | 51 |
| 33 cases of cancer of the stomach, with after-history .. .. .. .. | 24 |
| 41 cases of gastric or pyloric ulcer, with after-history .. .. .. .. | 35 |
| 12 cases of symptoms, but no ulcer found with after-history .. .. .. | 10 |
| 6 cases of duodenal ulcer, with after-history .. .. .. .. .. | 5 |
| 7 cases of hour-glass stomach, with after-history .. .. .. .. | 7 |
| 7 cases of hæmatemesis, with after-history .. .. .. .. .. | 6 |
| 2 cases of perigastric adhesions, with after-history .. .. .. .. | 1 |
| 1 case of congenital stenosis of pylorus, with after-history .. .. .. | 1 |

The two columns of this table present five more in the total than the figures given in the previous table, because five patients with perforated gastric ulcer treated by primary or secondary gastro-jejunostomy are counted twice.

The outstanding feature of the whole is the extraordinary value of gastro-intestinal short-circuiting for ulcer of the stomach or duodenum. A whole budget of letters was received speaking in terms of enthusiasm of the relief obtained. More than thirty patients had not a symptom to complain of, and could, as many of them said, " eat anything, and enjoy it." Most of these had previously spent months or years in continual misery, only partially alleviated by washing out, restricted diet, prolonged rest in bed, and medicines. Several of them write that " the operation has made a new man of me." It would be difficult to find any surgical proceeding in which the change from chronic distress and daily disability to perfect health is more marked or satisfactory. We shall have to speak later of some of the failures and dangers of gastric surgery, but these must not be allowed to obscure the remarkable benefits conferred on the great majority of the sufferers. One cannot help thinking that in many of the cases the symptoms had been allowed to go on far too long.

Another remarkable feature is the occurrence of so many cases diagnosed clinically as gastric ulcer, but presenting no signs of the condition on the operation table. This relates not only to five patients with severe hæmatemesis, but also to a dozen others, whose symptoms had been indistinguishable from those of genuine organic trouble. In two of them the stomach was notably dilated. It is hard to explain why they suffer so severely, and we are not yet able to make a diagnosis before operation, which, however, does not matter much, because even in these cases gastro-jejunostomy generally brings relief.

A third point of interest is the association of gastric ulcer, or symptoms of gastric ulcer, with movable kidney. Of thirty cases of movable kidney treated by operation at the Infirmary during ten years, three also appear in the present lists, and a fourth has been treated in a medical ward for gastric ulcer since her kidney was fixed. In one case (A. M.) the kidney was fixed twice, without relief, before the girl was finally cured by gastro-jejunostomy. In another (E. K.) a patient two years after kidney fixation was admitted dying of perforated gastric ulcer. In the third case (K. C.) the nephrorraphy was completely successful for years, and then a gastric ulcer formed. In a fifth case (F. A.) no operation was performed on the kidney, but it was very freely mobile when the patient was under treatment for stomach symptoms. It is possible that the reflex hyperchlorhydria which is produced by almost any chronic abdominal condition is responsible for the formation of the ulcer.

### PERFORATED GASTRIC OR DUODENAL ULCER.

There have been 61 cases of perforated gastric or duodenal ulcer; in two of these no operation was performed (G. P. was not diagnosed, and L. M. was admitted dying); in one (N. W.) the ulcer could not be found after prolonged search. These three cases all died. Of the remaining 58 ulcers closed by operation 26 died. The operative mortality is therefore 44 per cent.

*Age.*—The youngest patients were 17, the oldest was 77.

| | | | | |
|---|---|---|---|---|
| Between | 17 and 20—12 | gastric | cases, | 0 duodenal. |
| ,, | 21 and 30— 9 | ,, | ,, | 4 ,, |
| ,, | 31 and 40—17 | ,, | ,, | 1 ,, |
| ,, | 41 and 50— 6 | ,, | ,, | 0 ,, |

Over 50—5 gastric cases, 1 duodenal.

It will be seen that all ages from 17 to 40 are about equally liable, and that there is no marked difference in the age distribution of perforated gastric or duodenal ulcers. There is not the great preponderance of girls about 20 that the books lead one to expect.

*Sex.*—Of gastric or pyloric cases, 23 were male and 26 female. Of duodenal cases, 4 were male and 2 female.

*Previous History of Stomach Symptoms.*—In 3 cases there had been no warning symptoms at all. In 11 cases there had been mild dyspepsia, not sufficient to cause invalidism, for some weeks or months. In 18 cases there had been very definite evidence of gastric ulcer, such as continual pain, vomiting, or hæmatemesis. In 2 cases there was noted a relation to over-distension of the stomach. One girl (H. W.) had had a large supper of meat and punch, and a man (H. P.) perforated after taking a whole syphon of soda water with whisky to correspond.

*Situation of Perforation.*—In 33 cases the perforation affected the anterior wall of the stomach. In 11 cases the perforation affected the posterior wall of the stomach. In 8 cases the perforation affected the pylorus itself, in front. In 6 cases the perforation affected the duodenum.

For some reason or other the pylorus proved to be much the most favourable site for recovery. Only one of eight cases died. Five of the six duodenal patients recovered.

**The Operation Mortality.**—Three factors influencing the mortality may be mentioned :—

1. *The nature of the operation.*

Three patients were treated by closure of the ulcer and immediate gastro-jejunostomy. Of these two recovered and one died (D. S., E. K., H. P.).

Four patients had the ulcer excised ; in two of these the incision was sewn up in the manner of a pyloroplasty. These both recovered, the other patients died (F. J., T. P., H. G., G. C.).

In one case the ulcer could not be found, and the patient died (N. W.).

The remaining cases were treated by simple suture, with or without an omental graft. Of 13 cases in which the intestines were swilled over with saline fluid, 7 recovered and 6 died ; of cases in which the exudate was mopped out but no swilling was used, 21 recovered and 11 died.

2. *The Time Factor.*—In 47 patients there was a very sudden onset, the pain coming on in an instant with awful severity ; in 9 cases the symptoms crept on by degrees.

Dealing with the acute cases first :—

Operation within 12 hours—19 recovered, 5 died.

Operation from 12 to 24 hours—4 recovered, 6 died.

Operation over 24 hours—3 recovered, 10 died.

These figures emphasise the importance of early operation.

Of the chronic cases, none were operated on in less than 24 hours. 6 recovered and 3 died.

3. *The Site of the Perforation.*—Pyloric and duodenal perforations were by far the most favourable, presumably because they allow of less escape of gastric contents.

The common cause of death was of course peritonitis and shock (11 cases). In two cases the perforation burst open again, and one each of the following fatal calamities occurred: double pneumonia, fluid in chest, abscess in chest, pulmonary embolism, perisplenic abscess, leaking gastro-jejunostomy wound, intestinal obstruction by adhesions, a second perforation at another site, pyiephlebitis, subphrenic abscess, vagus inhibition by chloroform during a second operation for chest trouble.

**The After-History of the Recovered Patients.**—Of the recoveries, 23 have been followed and 10 lost sight of.

Of these 23, two (D. S., E. K.) had a primary gastrojejunostomy performed, and are both absolutely cured, and can eat anything without trouble.

Two had the ulcer excised and the incision sewn up like a pyloroplasty. One (T. P.) is completely cured, and the other (F. J.) can take ordinary food, but thirteen months after the operation began to suffer from occasional pain after meals. She does not vomit.

Of the remaining 19, only four express themselves as quite cured. None of these had had much dyspepsia before the perforation; two of them had had no symptoms at all. Eleven patients still suffer from indigestion or vomiting; they are not usually incapacitated from work, but are liable to bad attacks periodically. Two patients came in again for a gastro-jejunostomy. One of these is completely cured (F. H.), the other is considerably improved (G. M.).

One patient (D. H.) has an enormous incisional hernia, which has been operated on unsuccessfully, and he is in continual misery.

**Remarks and Conclusions.**—There are certain differences between the gastric ulcer that perforates and the gastric ulcer that causes chronic indigestion. The average age is about ten years younger. There may be very trifling symptoms or none at all. In 85 per cent. of the cases that perforate the ulcer is not on the pylorus. In 52 per cent. of the cases that come to operation for dyspepsia the ulcer is on the pylorus.

There is not much difference in the incidence of this catastrophe on the two sexes, and it is equally common at any age from 17 to 40.

Early operation, within twelve hours of perforation, saves about 80 per cent. of the patients ; later operation is usually unavailing, but the results do not get very bad until the second day. Cases that begin gradually are more favourable for late operation. Even if the patient does not succumb to shock and peritonitis there are many other possible complications.

With regard to the nature of the operation to be performed, our figures tell decidedly against the practice of employing lavage for the intestines.

Nearly all the sufferers continue to complain of indigestion afterwards, though it does not seem to be very severe. This is an argument for doing gastro-jejunostomy at the time if the patient will stand it. The results then become very satisfactory.

### CANCER OF THE STOMACH.

Thirty-three cases of cancer of the stomach have been treated, including 25 men and 8 women. The ages vary from 39 to 71, the great majority being between 48 and 60.

Only in four patients was there a history of gastric troubles eighteen months in duration; usually the symptoms had come on within less than a year. Pain was always present, vomiting was usual, and there is nearly always a note of anorexia and wasting. Only three patients had vomited blood, and in one of these it was eighteen months before admission. Obvious or occult blood in the stools was much commoner. A lump was felt in the abdomen in 19 cases out of the 33, and the stomach was often much dilated. There is no note on the chemical examination of the vomit in 11 cases, in 1 the hydrochloric acid was normal, in 3 it was deficient, and in 7 it was absent.

Great difficulty was experienced on several occasions in making a definite diagnosis of malignancy even after opening the abdomen. The after-history of two persons (E. T., M. B.) sent out with a gastro-jejunostomy for supposed cancer suggests strongly that they were cases of ulcer; one of them is nowperfectly well in every way, although the operation was early in 1907. Another patient (W. B.) was submitted to pylorectomy on the view that the mass was cancerous; it proved on microscopical examination to be simple, and unfortunately the man died of shock. It is possible, but not certain, that the ulcer may have been malignant in a woman of 25 who died of diarrhœa one year after gastro-jejunostomy for what was taken to be a large inflammatory mass (F. L.). In another case (M. P.), now lost sight of, it was left in doubt what the nature of the mass in the pylorus might be.

*Situation of the Growth.*—In 16 cases the pylorus was affected, in 13 the growth occupied the body of the stomach or the curvatures, in 3 it invaded the cardiac end.

**Operative Treatment and its Results.**—In 20 cases the abdomen was explored and closed again. Of these 7 patients left the Infirmary and are lost sight of. Five others died within a month, but no one seems to have been hastened out of the world by the operation. Only one individual lived for more than four months. This one (H. L.) presented a remarkable history. He was a man of 56, who had been suffering from dyspepsia and hunger-pain with vomiting for many years. On exploration a mass as large as an orange was observed at the cardiac orifice, and nothing was done. He was still alive two and a half years after, and had another operation, when a huge irremovable mass was found. Shortly after this he died. It is possible that the original condition was a simple ulcer.

In 8 cases a gastro-jejunostomy was performed. Two patients died of the operation within a few days. Another obtained no relief, and was then lost sight of. Of the other five, four seem to have obtained some measure of transient comfort, the fifth died in the train on the way home six weeks later. One patient lived five months; he had flatulence and pain, but no vomiting. Another improved so much that when he left I wrote, "Astonishingly well, gained 34lb. Not an unpleasant symptom since, so that one doubts the diagnosis." He died nine months after in "excruciating pain." Another was "fairly well for two months," but then got bad again, and died in five months. The remaining patient did well whilst in the Infirmary, and gained nearly four stone in as many months. She has since been lost sight of.

In four patients an extensive resection was performed. Two died of shock within a few days. Another (E. H.) recovered excellently, and was very well for some months, then the growth recurred and she died nine months after. The fourth patient (S. L.) was operated on in 1908, a gastro-jejunostomy being performed first and a partial gastrectomy one week later. The malignant nature of the growth was confirmed by the microscope. He was greatly relieved for two years, and in July, 1910, was hale and hearty, but since then there has been continual pain with some vomiting and hæmatemesis. An exploratory laparotomy has lately been performed, and a recurrence was found.

### GASTRIC OR PYLORIC ULCER.

Forty-one cases fall under this heading, excluding those patients in whom nothing was found at operation. The hour-glass stomachs and operations for severe hæmatemesis are also put under other headings. All the patients in this group, therefore, were submitted to operation for pain or vomiting, with or without evidence of dilatation of the stomach. If there had been vomiting of blood, it was only as an incident in the course of other symptoms, and not the prime cause for interference. Hydrochloric acid was never absent from the gastric contents; it was sometimes deficient and sometimes excessive.

In many of the cases there was a long history of continual misery. Many of them had undergone every variety of treatment, including dieting, rest in bed, and regular lavage, with little or no relief. One or two of them had already been operated on, one had had her kidney fixed twice and her appendix removed, another had had an appendicectomy.

**Age and Sex.**—There were 13 men and 28 women.

| | | |
|---|---|---|
| Under 20 | .. .. .. .. | 2 cases. |
| Between 21 and 30 | .. .. .. | 8 ,, |
| ,, 31 and 40 | .. .. .. | 10 ,, |
| ,, 41 and 50 | .. .. .. | 15 ,, |
| Over 50 | .. .. .. .. | 5 ,, |

**Situation of the Ulcer.**—Twenty of the ulcers were situated at the pylorus, eighteen affected the body of the stomach, and in two there were multiple ulcers.

**Operative Treatment and End-Results.** One patient (E. L.) was explored when in a dying condition, and nothing was done.

*Excision of Ulcer.*—This was practised three times. In one case (W. B.) an extensive pylorectomy was done in the belief that the mass was cancerous, and the patient died. Another (T. R.) has not been improved at all by the operation, and has been explored again in another hospital without benefit. The third (E. W.) is greatly improved, but not quite free from symptoms; she can usually take ordinary food.

*Gastro-duodenostomy.*—In four cases an opening has been made between the stomach and the duodenum. One is absolutely cured (P. R.). In another case (T. B.) there was some relief for six months, but since then he has been as bad as ever, and lives on bread and Benger's food. The other two have been lost sight of ; one of them promised well, the other did not.

*Gastro-jejunostomy.*—This operation was performed in 33 cases. The posterior anastomosis was always undertaken. In at least three patients a long isoperistaltic loop was made, and in three others an isoperistaltic short-circuiting with no loop. All except one in the former class, who has relapsed, have been perfectly cured. In the great majority of the cases the antiperistaltic no-loop operation was done.

Taking the results all together, there have been no deaths from the operation, but two patients have died since. One of these (H. H.) was perfectly well for six years, and then after a fortnight's pain and vomiting brought up a quart of blood. A fortnight later he bled into the bowel and died. There was no autopsy. The other patient (F. L.) did fairly well for a year, and then died with diarrhœa lasting two months. There was no autopsy. It is possible that this was a cancer.

Two patients (K. C., M. B.) only show slight improvement, although they were relieved for a month or two at first.

Five patients show great improvement, lasting from one to four years, but they have a little occasional pain or vomiting. In two of them the symptoms are really very trifling, and they can all do their work. In one case at least the trouble is due to the fact that the patient is quite edentulous. He was perfectly well for four years. [1]

Twenty patients out of twenty-nine followed through are absolutely and perfectly cured up to the present, and can take any ordinary food.

Four patients have been lost sight of.

SYMPTOMS OF GASTRIC ULCER: NO ULCER FOUND.

Included under this heading are cases of pain or vomiting diagnosed as gastric ulcer in which nothing, or only a little doubtful thickening of the pylorus, was found on the table. They are 12 in number, of which 2 have been lost sight of. In two cases gastro-duodenostomy was performed. One (A. S.) is quite cured. The other (C. G.) was relieved for nine months, but now gets much severe pain in attacks about twice a year, and her diet is strictly limited.

Another patient (F. A.), with some gastroptosis, had Finney's operation performed. After twenty-four hours she was given peptonised milk, and at once commenced to vomit blood until she died sixteen hours later. Nothing was found to account for it at the *post-mortem*.

The remaining 9 patients were treated by a posterior gastro-jejunostomy. Of 7 followed through, 1 is quite free from

[1] Another has just been explored for an acute attack of pain, but nothing was found to account for it.

symptoms (for four years), 3 are considerably improved but not free from symptoms, and 3 are no better. One (E. G.) of these is suffering from locomotor ataxia, and is now insane. Another (J. S.) was relieved for a while, but has been bad since; she had her appendix removed, and is now rather better, but still an invalid. One of those only partially relieved has tertiary syphilis, which may account for some of his troubles.

It will be seen that the results in this class are not so favourable as when a definite ulcer was discovered. Two are cured and four improved out of ten followed through. In the previous class, of 31 patients treated by a short-circuiting operation and followed through 21 are absolutely cured and 5 greatly improved.

### DUODENAL ULCER.

Of 6 cases, 5 were men and 1 a woman. All except one were over 30.

There was evidence on which a diagnosis might have been made in several of the cases. In three out of the six the pain was late after food, bore no relation to meals, or was relieved by eating. This sign must not be trusted too implicity, however, as five patients with pyloric ulcer presented the same symptom, and so did one girl with no ulcer at all. Three of the cases of duodenal ulcer had vomited blood, and one had had melæna.

One of the patients was treated by gastro-duodenostomy, but is lost sight of.

The other five had a gastro-jejunostomy performed. One (A. R.) died suddenly and unexpectedly on the sixth day. At the autopsy there was perihepatitis, pericarditis and œdema of the lungs, but the notes are inadequate. Three patients are perfectly cured, and one (J. V.) is almost well. His only trouble is that he gets pain and vomiting after stooping and digging (he is a country policeman). He also finds that he must avoid cooked vegetables.

### HOUR-GLASS STOMACH.

Seven patients have been treated for this condition. Six were female and one male. Their ages ranged from 22 to 42, most were about 35. All had been suffering from gastric symptoms for many years—one said 10 years, another 21, another 15, and a fourth had been washing out his stomach for 6 years. This long history is of great importance in making a diagnosis. Four had had severe attacks of hæmatemesis at various times. In one case the condition was recognised before operation by the help of a very conclusive skiagram after a bismuth meal.

The general results are as follows :—

3 cases perfectly cured.
1 case much improved.
1 case not improved.
2 cases died.

Of the patients who are cured, one had a double gastro-jejunostomy performed, another had a gastroplasty of the constriction and a gastro-jejunostomy of the distal pouch, and the third had a gastro-jejunostomy of the proximal pouch.

The patient much improved (A. H.) had a duodenal ulcer as well as hour-glass stomach. Gastroplasty of the constriction and gastro-jejunostomy of the proximal loop were performed.

The patient who is no better (G. B.) had a pyloric ulcer contracting, and the hour-glass constriction was only moderate. A single gastro-jejunostomy was performed.

Both the patients who died had a gastro-jejunostomy. One of them, who suffered from Graves's disease, died of perforated gastric ulcer some days after. The other developed pneumonia.

### HÆMATEMESIS.

Included under this heading are seven patients in whom the indication for the operation was severe bleeding. Where bleeding was only one of other indications the cases are classed elsewhere. In one (G. K.) of these seven individuals severe hemorrhage came on three weeks after operation for a perforated gastric ulcer. In the others there had been repeated hemorrhages.

In two cases an ulcer was present in the stomach, but nothing abnormal could be found in the stomach or duodenum of five of these patients. It is plain that severe repeated hæmatemesis is not an evidence of gastric ulcer. Three of the patients had been treated for pain and a little vomiting, in the others there was scarcely any warning before the bleeding came on.

With the exception of one recent case, one which has a persistent sinus after gastro-duodenostomy, all the patients are cured, although one has some nausea and a feeling of tingling in the scar. These five had gastro-jejunostomy performed. Two of them have been followed for four years. One has been lost sight of.

### PERIGASTRIC ADHESIONS.

Two patients had dilated stomachs with adhesions about the pylorus. Both were treated by gastrojejunostomy. One died a month later of bronchiectasis. The other was much improved at first, but is now lost sight of. It is possible that there may have been ulceration in these cases, although no scar was found. One had had hæmatemesis.

### CONGENITAL STENOSIS OF PYLORUS.

One infant, aged six weeks, was treated for this condition. He suffered from constipation and forcible vomiting. There was visible gastric peristalsis, and the pylorus was felt once. He lost a pound in a fortnight in spite of washing out and treatment with tincture of opium. At the operation a very thickened pylorus was found and submitted to pyloroplasty. For three weeks all went well, and the weight increased by six ounces; then came a period of standstill, and the child died for no apparent reason.

### TABLE OF RESULTS.

| Disease. | Operation. | Total Cases. | Lost Sight of. | Cases followed. | Quite Cured. | Much Improved. | Severe Symptoms still. | Died since leaving. | Died before leaving. |
|---|---|---|---|---|---|---|---|---|---|
| Perforated Gastric Ulcer .. | All operations .. .. .. .. | 53 | 9 | 44 | 7 | 8 | 4 | 0 | 25 |
| ,, .. | Suturing, drainage .. .. .. | 47 | 9 | 38 | 4 | 8 | 4 | 0 | 22 |
| ,, .. | Excision of ulcer .. .. .. | 3 | 0 | 3 | 1 | 0 | 0 | 0 | 2 |
| ,, .. | Immediate gastro-jejunostomy | 3 | 0 | 3 | 2 | 0 | 0 | 0 | 1 |
| Perforated Duodenal Ulcer.. | All operations .. .. .. .. | 6 | 1 | 5 | 0 | 4 | 0 | 0 | 1 |
| ,, .. | Suture, drainage .. .. .. | 5 | 1 | 4 | 0 | 3 | 0 | 0 | 1 |
| ,, .. | Excision, pyloroplasty .. .. | 1 | 0 | 1 | 0 | 1 | 0 | 0 | 0 |
| Cancer of Stomach .. .. | All operations .. .. .. .. | 33 | 9 | 24 | 0 | 1 | 0 | 17 | 6 |
| ,, .. .. | Exploration only .. .. .. | 20 | 7 | 13 | 0 | 0 | 0 | 11 | 2 |
| ,, .. .. | Gastro-jejunostomy .. .. | 8 | 1 | 7 | 0 | 0 | 0 | 5 | 2 |
| ,, .. .. | Partial gastrectomy .. .. | 4 | 0 | 4 | 0 | 1 | 0 | 1 | 2 |
| ,, .. .. | Gastrostomy .. .. .. .. | 1 | 1 | 0 | 0 | 0 | 0 | 0 | 0 |
| Gastric Ulcer .. .. .. | All operations .. .. .. .. | 41 | 6 | 35 | 21 | 6 | 4 | 2 | 2 |
| ,, .. .. .. | Excision .. .. .. .. .. .. | 3 | 0 | 3 | 0 | 1 | 1 | 0 | 1 |
| ,, .. .. .. | Gastro-duodenostomy .. .. | 4 | 2 | 2 | 1 | 0 | 1 | 0 | 0 |
| ,, .. .. .. | Gastro-jejunostomy .. .. | 33 | 4 | 29 | 20 | 5 | 2 | 2 | 0 |
| ,, .. .. .. | Exploration .. .. .. .. | 1 | 0 | 1 | 0 | 0 | 0 | 0 | 1 |

| | | | | | | | | | |
|---|---|---|---|---|---|---|---|---|---|
| Symptoms of Ulcer; none found | All operations .. .. .. .. | 12 | 2 | 10 | 2 | 4 | 3 | 0 | 1 |
| ,, | Gastro-duodenostomy .. .. | 3 | 0 | 3 | 1 | 1 | 0 | 0 | 1 |
| ,, | Gastro-jejunostomy .. .. | 9 | 2 | 7 | 1 | 3 | 3 | 0 | 0 |
| Duodenal Ulcer .. .. .. | All operations .. .. .. .. | 6 | 1 | 5 | 3 | 1 | 0 | 0 | 1 |
| ,, .. .. .. | Gastro-duodenostomy .. .. | 1 | 1 | 0 | 0 | 0 | 0 | 0 | 0 |
| ,, .. .. .. | Gastro-jejunostomy .. .. | 5 | 0 | 5 | 3 | 1 | 0 | 0 | 1 |
| Hourglass Stomach .. .. | All operations .. .. .. .. | 7 | 0 | 7 | 3 | 1 | 1 | 0 | 2 |
| ,, .. .. | Single gastro-jejunostomy .. | 4 | 0 | 4 | 1 | 0 | 1 | 0 | 2 |
| ,, .. .. | Double gastro-jejunostomy .. | 1 | 0 | 1 | 1 | 0 | 0 | 0 | 0 |
| ,, .. .. | Gastroplasty and gastro-jejunostomy | 2 | 0 | 2 | 1 | 1 | 0 | 0 | 0 |
| Hæmatemesis .. .. .. | All operations .. .. .. .. | 7 | 1 | 6 | 4 | 2 | 0 | 0 | 0 |
| ,, .. .. .. | Gastro-duodenostomy .. .. | 1 | 0 | 1 | 0 | 1 | 0 | 0 | 0 |
| ,, .. .. | Gastro-jejunostomy .. .. | 6 | 1 | 5 | 4 | 1 | 0 | 0 | 0 |
| Perigastric Adhesions, Dilated Stomach | Gastro-jejunostomy .. .. | 2 | 1 | 1 | 0 | 0 | 0 | 1 | 0 |
| Congenital Stenosis of Pylorus | Pyloroplasty .. .. .. .. | 1 | 0 | 1 | 0 | 0 | 0 | 0 | 1 |

### SUMMARY OF CASES.

### PERFORATED GASTRIC OR DUODENAL ULCER.

| Year. | Initials. | Age. | Sex. | Symptoms. | Time of Operation. | Findings and Treatment. | End-Result. |
|---|---|---|---|---|---|---|---|
| 1902 | A. C. | 30 | F. | Hæmatemesis 3 months ago. Acute onset of perforation | 4 hours .. | Anterior wall. Sewn up, drained | Recovered. Lost sight of. |
| ,, | N. W. | 16 | F. | Indigestion for months. Acute onset | 23 hours | Perforation could not be found | Died 12th day. |
| 1903 | W. C. | 40 | M. | Sudden onset. Very ill .. .. | 15 hours.. | Ant. wall. Sewn up, drained | Died under 24 hours. |
| ,, | B. S. | 20 | F. | Three months dyspepsia. Acute onset | 2 days .. | ,, ,, | Recovered. Lost sight of. |
| ,, | M. S. | 20 | F. | Recently in medical ward for gastric ulcer. Acute onset | 24 hours.. | Ant. wall sewn up. Swilled, drained | Recovered. Lost sight of. |
| ,, | L. C. | 18 | F. | Dyspepsia for years; vomited blood. Acute onset | 2 days .. | Ant. wall. Sewn up, drained | Died 3rd day. Perisplenic abscess. |
| ,, | A. P. | 37 | M. | Dyspepsia 1 year. Acute onset | 8 hours.. | Ant. wall. Sewn up, swilled, drained | Seen 4.1911. Dyspepsia ever since, loses work by it. |
| ,, | E. N. | 19 | F. | Dyspepsia always. Acute onset | 6 hours.. | Ant. wall. Sewn up, drained | Recovered, Lost sight of. |
| ,, | B. H. | 17 | F. | Occasional dyspepsia. Gradual onset at Wells | 3 days .. | Post. wall. Sewn up, swilled, drained | Died 3rd day. Perforation reopened. |
| 1904 | J. W. | 25 | M. | Ill for 8 weeks with subphrenic abscess | 3 days .. | Abscess opened .. .. .. | Died 6 weeks later. Perforated duodenal ulcer. |
| ,, | E. H. | 18 | F. | Dyspepsia, worse lately. Sudden onset | 3 days .. | Ant. wall. Sewn up, drained | Died 12th day. Abscess in chest. |
| ,, | E. S. | 18 | F. | Dyspepsia 14 days. Acute onset | 27 hours.. | Ant. wall. Sewn up, swilled, drained | Died under 24 hours. |

| | | | | | | | |
|---|---|---|---|---|---|---|---|
| ,, | F. A. | 27 | M. | Slight dyspepsia 1 year. Acute onset | 8 hours . . | First part of duodenum. Perforated, sewn up, drained | Writes, 4.1911 : " For 3 years pain and vomiting ; now better." |
| 1905 | E. H. | 41 | F. | Dyspepsia 1 year, worse lately. Acute onset | 11 hours . . | Ant. wall. Sewn up, drained | Died. |
| ,, | H. S. | 21 | M. | Dyspepsia 1 year, worse lately. Acute onset | 3 hours . . | Duodenal. Sewn up, drained | Recovered. Lost sight of. |
| ,, | R. K. | 31 | F. | Severe dyspepsia for years. Acute case | 2 days . . | Post wall. Sewn up, swilled, drained | Recovered. Lost sight of. |
| ,, | G. C. | 45 | M. | Dyspepsia for years. Acute onset at Avonmouth | 3 hours . . | Post. wall. Sewn up, swilled, drained | Recovered. Lost sight of. |
| ,, | E. A. | 23 | F. | Pain and vomiting for months. Acute onset | 3 days . . | Appendicectomy. Huge perforation ant. wall. Sewn up, drained | Died under 24 hours. Another perforation of post. wall. |
| ,, | M. P. | 40 | F. | Pain and vomiting for years, worse lately. Sudden onset whilst lifting a patient | 8 hours . . | Ant. wall. Sewn up, swilled, drained | Writes, 4.1911 : " Relief for $1\frac{1}{2}$ years." Still attacks of pain and vomiting. Well in the intervals. |
| ,, | F. P. | 23 | M. | Dyspepsia 2 years. Sudden onset | ? | Ant. wall. Sewn up, swilled, drained | Died 15th day of pulmonary embolism. |
| ,, | F. K. | 48 | F. | Old case of nephrorrhaphy, recently vomiting. Sudden onset | 1 day . . | Post. wall. Sewn up, swilled, drained. | Died 4th day. |
| 1906 | T. C. | 40 | M. | Dyspepsia 2 years. Sudden onset | 3 hours . . | Post. wall. Sewn up, swilled, drained | Recovered. Lost sight of. |
| ,, | G. P. | 77 | M. | Acute pain and retention ; thought to be prostatic case | | No operation . . . . . . | Died under 24 hours. |
| ,, | R. E. | 35 | M. | Occasional dyspepsia. Sudden onset | ? | Post. wall on pylorus. Sewn up, drained | Died 7th day. Chloroform death. Abscess formed in right chest. |
| ,, | B. T. | 22 | F. | Pain and vomiting 2 months. Sudden onset | 12 hours . . | Post. wall. Sewn up, swilled, drained | Twice vomited blood. Recovered. Lost sight of. |
| ,, | E. T. | 17 | M. | From the medical floor. Sudden onset | ? | Ant. wall. Sewn up, swilled, drained | Died next day. |
| ,, | V. F. | 25 | M. | Some pain 1 hour after food ; vomiting. Acute onset | 3 hours . . | Ant. wall. Omental graft. swilled, drained | Died 5 weeks later. Fluid in chest. |

| Year. | Initials. | Age. | Sex. | Symptoms. | Time of Operation. | Findings and Treatment. | End-Result. |
|---|---|---|---|---|---|---|---|
| 1907 | H. T. | 37 | M. | Considerable pain and fulness after food. Acute onset | 24 hours.. | Perforated on pylorus. Sewn up, drained | Writes, 4.1911 : "Gets cramp pains across the stomach at intervals ; well between. Takes ordinary food. Feeling of fulness sometimes." |
| ,, | J. P. | 36 | M. | Colicky pain for fortnight, worse last 2 days | Gradual | Duodenal. Sewn up, drained | Writes, 4.1911 : "Operation gave real relief, but still get vomiting and pain at times ; not very often." Takes ordinary food. |
| ,, | L. M. | ? | F. | Admitted very ill and collapsed | No operation | .... | Died under 24 hours. Two ulcers, one perforated. |
| ,, | F. J. | 21 | F. | Little dyspepsia lately, vomited blood. Sudden onset | ?8 hours.. | Duodenal. Excised, sewn up, as pyloroplasty ; omental graft. Drained | Writes, 4.1911 : "Real relief for 13 months. Since then some pain, no vomiting. Takes ordinary food." |
| ,, | D. S. | 22 | F. | Pain and vomiting for 4 years. Acute onset | 12 hours. . | Ant. perforation, old adhesions. Sewn up. Gastro-jejunostomy. Drained | Got pneumonia and white leg. Writes, 4.1911 : "Operation a perfect success in every way." No symptoms, eats anything. |
| ,, | M. W. | 19 | F. | Dyspepsia 3 weeks. Sudden onset | ? | Ant. wall. Sewn up, drained | Mistress writes, 4.1911 : "Operation entirelysuccessful." Lately lost sight of. |
| ,, | R. T. | 20 | F. | Dyspepsia 3 months. Acute onset | 4 days .. | Ant. wall. Sewn up, drained | Died next day. |
| 1908 | L. S. | 35 | F. | Dyspepsia for years. Acute onset. Dying | 2 days .. | Ant. wall . Sewn up, drained | Died under 24 hours. |

| | | | | | | | |
|---|---|---|---|---|---|---|---|
| ,, | F. P. | 27 | F. | Dyspepsia 3 years. Sudden onset | 6 hours.. | Duodenal, near pylorus. Sewn up, omental graft, drained | Writes, 4.1911: "Great relief." No pain or vomiting, occasional slight indigestion. Takes ordinary food. |
| ,, | A. H. | 37 | M. | No previous symptoms. Acute onset. Very ill | 3 days .. | Perforation on pylorus. Sewn up, drained | Writes, 4.1911: "Operation successful." No symptoms. Takes ordinary food. |
| ,, | H. G. | 36 | F. | Invalided out of army for gastric symptoms 2 years ago. Onset acute | 24 hours.. | Ulcer excised. Drained .. | Died under 24 hours. |
| ,, | E. L. | 20 | F. | No previous symptoms. Not very ill | 3 days .. | Ant. wall. Perforation sealed by omentum and lymph. Not much peritonitis. Sewn up, drained | Writes, 4.1911: "Enjoys splendid health." Takes ordinary food, no trouble at all. |
| ,, | T. P. | 62 | M. | Dyspepsia 10 years, once vomited blood. Pain 1½ hours after food. Has had renal calculi; operation 4 years ago. Sudden onset | 1 day .. | Perforation on pylorus. Excised, sewn up as pyloroplasty. Drained. Calculi in kidney | Writes, 4.1911: "Great relief from all stomach trouble." No symptoms or pain since. Takes ordinary food. |
| ,, | H. P. | 38 | M. | No previous dyspepsia. Sudden perforation after drinking a whole syphon of soda-water, with whisky | Under 24 hours | Ant. wall. Sewn up, drained | Died under 24 hours. |
| 1909 | W. K. | 59 | M. | Pain 1½ hours after food for years. Sudden onset | ? 3 hours | Post. wall, near pylorus. Sewn up, drained | Died. Double pneumonia; wound burst open; fæcal fistula. |
| ,, | E. K. | 65 | M. | Periodical severe gastric pain, large vomits. Pyloric tumour. Thin. In great pain. Not suspected of having perforated | ? very recent | Pyloric ulcer perforated. No peritonitis. Sewn up, gastro-jejunostomy | Gained 17 lb. Writes, 4.1911: "No symptoms since operation. Eats anything." |
| ,, | A. M. | 22 | F. | Recent dyspepsia. Sudden onset, but able to get about a little since | 2 days .. | Perforation on pylorus. Sewn up, drained | Writes, 4.1911: "Operation gave great relief, am wonderfully well." A little indigestion still; is careful with diet. |

PERFORATED GASTRIC OR DUODENAL ULCER (*continued*).

| Year. | Initials. | Age. | Sex. | Symptoms. | Time of Operation. | Findings and Treatment. | End-Result. |
|---|---|---|---|---|---|---|---|
| 1909 | H. D. | 32 | M. | Dyspepsia for months. Acute onset | 1 day .. | Perforation near pylorus. Little peritonitis. Sewn up, drained | Writes, 4.1911: "Pain at times. Can eat most things." |
| ,, | E. P. | 41 | F. | Old in-patient with gastritis; has had gallstones. Now diagnosed as appendicitis | 3 days .. | Large perforation, ant. wall. sewn up, drained | Perforation burst open 3 days later. Second operation. Died next day. |
| ,, | H. W. | 21 | F. | Dyspepsia 6 months. Acute onset after large supper of meat and punch | ? 3 hours | Post. wall. No general peritonitis. Sewn up, drained | Writes, 4.1911: "Operation most successful." One attack of pain and vomiting since, occasional dyspepsia. Takes ordinary food. |
| ,, | D. H. | 35 | M. | Little previous dyspepsia. Acute onset after dinner | 10 hours.. | Ant. wall. Sewn up, drained | Incisional hernia; unsuccessful attempt at closure since. Seen, 4.1911. Huge incisional hernia, much pain and vomiting. Cannot work. Very careful of diet. |
| ,, | W. D. | 47 | M. | Dyspepsia for months. Acute onset | 24 hours.. | Ant. wall. Sewn up, drained | Died soon after. |
| ,,<br>(see un | F. H.<br>der Gas | 37<br>tric Ul | M.<br>cer) | Has had previous pain, vomiting and hæmatemesis. Acute onset | 5 hours.. | Ant. wall. Old adhesions. Sewn up, omental graft, drained | Pain and vomiting continued, could not work. Eventually cured by gastro-jejunostomy. |
| ,,<br>(see un | G. M.<br>der Gas | 32<br>tric Ul | M.<br>cer) | Pain 10 days ago. Acute onset | 3 hours.. | Perforation on pylorus. Sewn up, omental graft, drained | Pain and vomiting continued. Greatly relieved by later gastro-jejunostomy. |
| 1910 | F. P. | 58 | M. | Mild dyspepsia. Sudden onset | ? 8 hours | Duodenal. Sewn up, drained | Recovered. Lost sight of. |

| | | | | | | | |
|---|---|---|---|---|---|---|---|
| ,, | E. B. | 38 | F. | Pain and vomiting and careful dieting for years. Acute onset | Over 24 hours | Ant. wall. Sewn up, drained | Died 1 month later, after continual vomiting. Intestinal obstruction by adhesions of ileum to wound. Stomach perforated, and contents in lesser sac. No peritonitis. |
| 1910 (Nov.) | A. P. | 23 | F. | In Medical ward 7 days with vomiting. Temp. 102°. Leucocytosis. Explored, pus found | ? | Leaking gastric ulcer; pus between stomach and liver. Sewn up, drained | Writes, 4.1911: "Greatly benefited, much better." Two attacks of pain and vomiting since. Cannot eat meat. |
| 1910 | H. P. | 50 | M. | Dyspepsia many years. Sudden onset, awful pain | 16 hours.. | Rather hour-glass stomach, perforation ant. wall. Sewn up, gastro-jejunostomy of proximal pouch. Drained | Died 12 days later from leakage of the gastro-jejunostomy wound at the cardiac end. |
| 1910 (May) | A. P. | 18 | F. | Pain and vomiting 3 months. Acute onset | 24 hours.. | Ant. wall. Sewn up, drained | Writes, 4.1911: "Great benefit." Quite free from symptoms. |
| 1910 (May) | A. C. | 32 | M. | Pain and vomiting ½ hour after food. Is an out-patient. Acute onset | 2½ hours.. | Ant. wall. Sewn up, drained | Seen 4.1911. Went on well 4 months till he started work. Since then severe hunger-pain. Diet severely restricted. No vomiting. At work. |
| 1910 | H. M. | 30 | M. | (No notes.) | 11 hours.. | (No notes.) | Died next day. |
| 1910 | E. G. | 64 | F. | Dyspepsia 2 weeks. Sudden onset. Sent from Blagdon as appendicitis | ? | Perforation of pylorus. Sewn up, drained | Died 1 week later. No *post-mortem*. |
| 1910 | G. C. | 33 | M. | Sudden onset .. .. .. .. | 4½ hours.. | Ant. wall cardiac end. Excised, drained | 3 weeks later, rigors with leucocytosis. Died. *Post-mortem*, pylephlebitis. |
| 1910 (Jan.) (see under Hæmatemesis) | G. K. | 59 | M. | Dyspepsia for years. Gradual onset. Diagnosed as perforation into lesser sac | 3 days .. | Ant. pyloric perforation. No leakage | 22 days later a severe hæmatemesis. Quite cured by gastro-jejunostomy. |

| Year. | Initials. | Age. | Sex. | Symptoms. | Findings and Treatment. | End-Results. |
|---|---|---|---|---|---|---|
| 1902 | E. H. | 61 | F. | Pain, vomiting 7 months. Melæna. Very anæmic. Lost 4 st. Lump felt | Cancer of pylorus (confirmed by microscope). Partial gastrectomy with end-to-end suturing | Gained 24 lb. in 5 weeks; did well for several months. Died of recurrence 9 months later. |
| ,, | E. W. | 48 | F. | Pain 1 year, daily vomiting, wasting. Lavage, no relief | Large mass in pylorus, secondaries in bowel, big stomach. Gastro-jejunostomy | Complete relief for 4 months. Gained 3 st. 12 lb. Since lost sight of. |
| ,, | E. O. | 68 | F. | Pain, wasting, costive. Lump felt | Large cancer of cardiac end; secondaries. *Nil* done | Died 2 months after leaving. |
| ,, | C. B. | 51 | M. | Lump and dyspepsia 9 months. Severe pain, vomiting, wasting 3 months | Diffuse leather-bottle stomach. Extensive removal, end-to-end union | Died 5th day of shock. |
| ,, | W. C. | 49 | M. | Vomiting, pain, wasting. One attack hæmatemesis | Large inoperable growth. *Nil* done | Died 1 month later, vomiting to the end. |
| 1903 | E. T. | 49 | F. | Small vomits immediately after food, 10 months. Pain, wasting, lump | Gastrostomy .. .. .. .. .. | Recovered. Lost sight of. |
| ,, | J. H. | 54 | M. | From Medical ward. Vomiting 1 month, costive, wasting, lump in pylorus. Sarcinæ, lactic acid, HCl 0.12 % | Lavage; no relief. Explored. Large growth in pylorus. Gastro-jejunostomy | Died 4 days later. |
| 1905 | E. C. | ? | F. | Pain after food 3 years. Great flatulence. Lump felt | Extensive cancer. *Nil* done .. | Lost sight of. |
| ,, | J. D. | 54 | M. | Pain, anorexia, wasting; 2 months' history. Lump felt. Takes fluids only. Lactic acid, no HCl. | Extensive cancer. *Nil* done .. | Died 5 weeks after leaving, exceedingly emaciated. No pain or vomiting. |
| 1906 | A. W. | 39 | M. | Copious yeasty vomiting. Lost $4\frac{1}{2}$ st. Lump felt | Growth in pylorus; glands. Dilated stomach. Gastro-jejunostomy | A month later I wrote: "Astonishingly well, gained 34 lb. Not an unpleasant symptom since, so that one doubts the diagnosis." Died 9 months later "after excruciating pain." |

| | | | | | | |
|---|---|---|---|---|---|---|
| ,, | W. E. | 60 | M. | Vomiting, wasting, anæmic. Large hæmatemesis 18 months ago. Lump | Cancer of lesser curvature. *Nil* done | Died "soon after leaving." |
| ,, | J. P. | 44 | M. | 4 years' pain several hours after food. Now very frequent vomiting | Cancer of pylorus. *Nil* done .. | Died 3 months' after leaving. |
| ,, | H. L. | 56 | M. | Many years' hunger-pain. Now vomiting and anorexia. Nc lump | Mass size of orange in cardiac orifice. Gall-stones in bile-duct. *Nil* done | Remained in same condition for $2\frac{1}{2}$ years, when he was again explored, and a huge growth found. Died soon after. |
| 1907 | R. F. | 44 | M. | Periodical pain and vomiting 2 years. Now constant vomiting. Lactic acid, no HCl | Lump found in greater curvature. *Nil* done | Lost sight of. |
| ,, | G. P. | 53 | M. | Pain 8 months, losing weight. Only vomited once. Lump felt | Mass near pylorus. Large glands. *Nil* done | Died 23 days after leaving, with much pain and hæmatemesis. |
| ,, | N. L. | 49 | M. | Pain and vomiting 1 year. Wasted. Lump felt | Mass in pylorus. Gastro-jejunostomy attempted, man too ill | Died 1 month after leaving. |
| ,, | M. A. | 67 | F. | Pain, anorexia, vomiting, wasting 8 months. Lump. Lactic acid, no HCl | Extensive cancer of body of stomach. *Nil* done | Lost sight of. |
| ,, | W. L. | 71 | M. | Pain and wasting 4 months. Little vomiting. Enormous stomach. HCl present | Extensive growth. *Nil* done .. | Lost sight of. |
| 1908 | S. L. | 57 | M. | Pain after food, vomiting, constipation, loss of weight, anorexia for 12 months. Very thin. Lump size of golf ball. Lactic acid, HCl 0.14 % | Growth in pylorus (proved cancer by microscope). Gastro-jejunostomy, a week later partial gastrectomy (Mayo's method) | In July, 1910, was hale and hearty, free from symptoms, and had put on flesh. Since going downhill. Now gets constant pain and vomiting, and has brought up blood. Perhaps a recurrence. |
| ,, | L. M. | 40 | M. | Pain, vomiting, constipation 3 months. Lost 2 stone. Lump. HCl nearly absent, yeasts and lactic acid. Occult blood in stools | Extensive cancer, with glands. *Nil* done | Vomited till he died 8 weeks later. |
| ,, | J. P. | 63 | M. | Dyspepsia and wasting. Lump felt | Diffuse growth. *Nil* done .. .. | Lost sight of. |
| ,, | C. S. | 65 | M. | Weakness and pain, 4 months. Wasting. No HCl. Lump felt | Cancer of pylorus. Partial gastrectomy (Mayo), gastro-jejunostomy | Died on 3rd day. |

CANCER OF STOMACH (*continued*).

| Year. | Initials. | Age. | Sex. | Symptoms. | Findings and Treatment. | End-Results. |
|---|---|---|---|---|---|---|
| 1908 | J. W. | 46 | M. | Pain, anorexia and enormous vomits, 2 months. Large stomach. HCl 0.2 % | Mass in pylorus. Gastro-jejunostomy | Died 6 weeks after in the train going home. |
| ,, | F. E. | 51 | M. | Pain 10 months, vomited once .. | Large pyloric growth, glands. *Nil* done | Lost sight of. |
| 1909 | W. S. | 57 | M. | Dyspepsia many years. Recently worse; vomiting, anorexia, wasting, lump | Growth near pylorus. Gastro-jejunostomy | Died 5 or 6 months later. Flatulence, pain, but no vomiting. |
| ,, | M. P. | 39 | F. | Pain, vomiting for many years. In-patient 10 years ago for it. Three attacks hæmatemesis. Dilated stomach. Absorption slow | Mass in pylorus, nature uncertain. Large stomach. Gastro-jejunostomy | Not improved. Since lost sight of. |
| ,, | A. R. | 46 | M. | Pain and enormous vomits, 6 months. Large stomach. HCl deficient. No occult blood | Cancer of body of stomach. *Nil* done | Lost sight of. |
| 1910 | W. M. | 58 | M. | Pain, vomiting and very dilated stomach, 6 weeks. Wasting. Fed on slops | Cancer of pylorus, enormous stomach. Gastro-jejunostomy | Died 4 days later. Stomach still very large. |
| ,, | J. B. | 65 | M. | Pain and occasional vomiting, $1\frac{1}{2}$ years. Wasting. Free HCl present | Growth of pylorus invading liver. Gastro-jejunostomy | Took food well for 2 months. Much pain, vomited blood. Died 6 months after operation. |
| ,, | M. P. | 53 | F. | Vomiting, wasting, 5 months. No HCl. Occult blood in stools. Lump | Universal cancerous nodules. *Nil* done | Died on 13th day. |
| ,, | H. B. | 60 | M. | A little dyspepsia lately. Lump. | Cancer of stomach. *Nil* done .. | Died on 15th day of embolism. |
| ,, | J. P. | 48 | M. | Flatulence, occasionally vomiting. Losing weight (3 lb. in a week) | Cancer of stomach, with metastases. *Nil* done | Died 3 weeks after leaving. |
| ,, | J. H. | 67 | M. | Dyspepsia many years. Vomiting and wasting 1 year. Dilated stomach | Cancer of lesser curvature, enlarged glands. *Nil* done | Died 3 weeks after leaving. |

### GASTRIC ULCER.

(For two further cases, see D. S. and E. K. under Perforated Gastric Ulcer.)

| Year. | Initials. | Age. | Sex. | Symptoms. | Findings and Treatment. | End-Results. |
|---|---|---|---|---|---|---|
| 1902 | A. R. | 33 | M. | Pain and vomiting. Twice in Medical ward. 10 months' duration. Vomited 1 pint of blood 9 months ago. Dilated stomach | Pyloric ulcer, adhesions. Isoperistaltic gastro-jejunostomy c loop | Practically well till 1909. Seen, 4.1911. Now gets great pain, vomiting and hæmatemesis, but is able to work. Quite edentulous. |
| 1903 | H. H. | 40 | M. | Pain and vomiting 8 years. Wasting, weight 8 st. 4½ lb. Dilated stomach for months. Bed, fluids and lavage for weeks; no better | Pyloric constriction, big stomach. 2-inch gastro-jejunostomy, c loop | Quite well until 1909, then, after 2 weeks, pain and vomiting, brought up 2 pints of blood; 2 weeks later faintness, melæna and death. No *post-mortem*. |
| 1904 | H. P. | 40 | F. | Pain and vomiting 3 years, now incessant. Big yeasty vomits. Pylorus palpable. Visible gastric peristalsis, splash. HCl .124 % | Pyloric ulcer, ? cancerous. Gastro-jejunostomy, c loop | Much better at first. Since lost sight of. |
| 1905 | M. B. | 52 | F. | (No Notes.) Pain since she was 14. Now says her life was a misery before the operation, and that she would have died | Large ulcer near pylorus. Gastro-jejunostomy, c loop | Writes, 4.1911: "Perfectly well, can eat anything. I have great cuase to be thankful I had it done. Real lasting relief." |
| ,, | E. J. | 23 | F. | In misery 1 year with pain. Living on milk and bread. Occasional vomiting. Lump felt. HCl .23 %. Nutrients no relief | Large mass in pylorus. Gastro-jejunostomy, c loop | Relieved at first, then vomiting severely. Lost sight of. |
| ,, | E. C. | 54 | M. | Always dyspeptic. Pain 12 months, no relation to food. Frequent vomiting. Requires morphia. HCl .09 % | Marked thickening and adhesions of pylorus. Gastro-jejunostomy c loop | Lost sight of. |

GASTRIC ULCER (*continued*).

| Year. | Initials. | Age. | Sex. | Symptoms. | Findings and Treatment. | End-Results. |
|---|---|---|---|---|---|---|
| 1906 | F. C. | 20 | F. | Twice in Medical Ward (1903, 1906) for pain, vomiting, and hæmatemesis (once). Thickening of pylorus felt ; tender | Large mass about ulcer of pylorus. Gastro-jejunostomy | Writes, 4.1911 : "The operation has given me real relief, and I can now take ordinary food without trouble." No pain or vomiting. |
| ,, | A. E. | 43 | M. | Dyspepsia 12 years, constant pain and daily vomiting 6 weeks. Very wasted. HCl .19 %. Lavage gave slight relief | Thickened pylorus, dilated stomach. Gastro-jejunostomy | Gained 11 lb. in a month. Writes, 4.1911 : "Since the operation I have been *quite* well." No pain or vomiting ; takes any food. |
| ,, | E. L. | 27 | F. | Pain, vomiting and hæmatemesis 5 years. Great pain and vomiting 3 days ; tender. Pulse 120, getting worse. Jaundice. Diagnosed as bleeding on perforation. Admitted very ill | Explored, old ulcer found. Patient dying. *Nil* done | Died under 24 hours. |
| ,, | W. B. | 56 | M. | Pain 2 hours after food, frequent vomiting, wasting. Ill 1 year. HCl deficient, sarcinæ, lactic acid present | Diagnosed as cancer of the pylorus. Pylorectomy with end-to-end union | Died 1 hour later. Proved to be ulcer by microscope. |
| 1907 | K. S. | 46 | F. | Old Medical in-patient. Pain 1 hour after food, constant vomiting. Ill 12 months | Ulcer of lesser curvature. Gastro-jejunostomy, c loop | Writes, 4.1911 : "The operation has given real relief. I was never better in my life." No pain or vomiting. |
| ,, | J. R. | 49 | M. | Pain and vomiting 12 years ; now vomits everything. Lost 2 stone. Dilated stomach | Old ulcer of pylorus. Gastro-jejunostomy | Writes, 4.1911 : "Has given me permanent relief." No pain or vomiting, eats ordinary food. |

| | | | | | | |
|---|---|---|---|---|---|---|
| ,, | L. D. | 18 | F. | Pain and vomiting 3 years. Tender spot in epigastrium. Dilated stomach | Pyloric ulcer, great narrowing. Antiperistaltic, no-loop, gastro-jejunostomy | Gained 9 lb. in a month. Writes, 4.1911 : " No pain or vomiting since. Takes ordinary food. Has developed Graves's disease. |
| ,, | E. T. | 54 | F. | Pain 2 years, several times vomited blood. Frequent vomiting. HCl deficient | Lump in pylorus, suspected to be cancer. Antiperistaltic, no-loop gastro-jejunostomy | Writes, 4.1911 : " The operation was a perfect success." No pain or vomiting. " Can take any food and thoroughly enjoy it." |
| ,, | A. M. | 21 | F. | Pain, vomiting, dilated stomach for years. Has had appendicectomy, and kidney fixed twice | Scar in pylorus. Gastro-jejunostomy | Writes, 4.1911 : Perfectly well for $1\frac{1}{2}$ years, now gets vomiting about once in 2 months. Diets carefully, rests in bed when ill. Great relief on the whole. |
| 1908 | F. L. | 25 | F. | Pain and vomiting 3 years. Old in-patient. Weight 5 st. 7 lb. Lump felt | Mass near pylorus. Gastro-jejunostomy | Died a year later after diarrhœa for 8 weeks. |
| ,, | A. W. | 35 | F. | Pain and vomiting many years. No dilatation of stomach found | Pyloric stenosis. Antiperistaltic, no-loop gastro-jejunostomy | Writes, 4.1911 : " Real relief at once, thoroughly successful." Can eat anything. |
| ,, | T. B. | 41 | M. | Pain 2 hours after food for 3 years. Once vomited. Tender | Pyloric ulcer. Gastro-duodenostomy | Writes, 4.1911 : " Relieved for 6 months, pain since. Lives on bread and Benger's. Not improved. |
| ,, | T. N. | 46 | M. | Dyspepsia many years, now copious vomits. Tenderness. Great pain after meat | Ulcer of cardiac end. Gastro duodenostomy | Not much better. Lost sight of. |
| ,, | P. R. | 27 | F. | Pain just after food and vomiting 6 years. Hæmatemesis 3 years ago. Out-patient 4 years. Tenderness. Dilated stomach | Pyloric ulcer. Gastro-duodenostomy | Writes, 4.1911 : " I cannot tell how grateful I am for the operation. Perfectly cured." Takes any food without trouble. |
| ,, | T. S. | 48 | M. | Pain and hæmatemesis $1\frac{1}{2}$ years ago in General Hospital. Now pain and vomiting, with a little blood, 2 months | Ulcer near pylorus. Antiperistaltic, no-loop gastro-jejunostomy | Better at the time, since lost sight of. |

| Year. | Initials. | Age. | Sex. | Symptoms. | Findings and Treatment. | End-Results. |
|---|---|---|---|---|---|---|
| 1909 | H. T. | 27 | M. | Pain, especially at night, vomiting. 7 years ago had hæmatemesis. Had appendix removed. Stomach washed out, no benefit | Ulcer on pylorus. Antiperistaltic, no-loop gastro-jejunostomy | Writes, 4.1911: "Going on all right since." Takes ordinary food. No symptoms at all. |
| ,, | M. D. | 32 | F. | Vomiting and repeated hæmatemesis for 8 years. In various hospitals. Lavage no benefit. | Ant. wall scarred. Antiperistaltic, no-loop gastro-jejunostomy | Writes, 4.1911: "Real relief after many years of suffering." Eats anything. No symptoms except flatulence. |
| ,, | A. H. | 43 | F. | Pain, vomiting. In Westminster Hospital. Lavage no benefit. Very dilated stomach. Free HCl present, lactic acid, Oppler-Boas bacilli | Ulcers on pylorus, and greater curvature, Antiperistaltic, no-loop gastro-jejunostomy | Writes, 4.1911: "Real relief." Has no symptoms, and eats anything. |
| ,, | G. M. | 33 | M. | Perforated pyloric ulcer 6 months ago. Since had pain 1½ hours after food, vomited twice. Stomach not dilated | Antiperistaltic, no-loop gastro-jejunostomy | Writes, 4.1911: "Now better than for some considerable time, and still improving." No relief at first. Fulness after meals, especially if large. Avoids meats. |
| ,, | E. E. | 49 | F. | Dyspepsia 3 years. Vomited blood 1 year ago, and again yesterday | Cardiac ulcer. Antiperistaltic, no-loop gastro-jejunostomy | Writes, 4.1911: "I do not vomit or get any pain worth speaking of." Care in diet. "Real relief." Still improving. |
| ,, | F. H. | 36 | M. | Perforated ulcer of ant. wall 10 months ago. Since vomiting, losing flesh, a little pain | Antiperistaltic, no-loop gastro-jejunostomy | Seen, 4.1911. No stomach symptoms since. Well nourished. Eats anything. Has lumbago. |
| ,, | E. W. | 42 | F. | Dyspepsia for years, severe pain 3 weeks. Lump felt, stomach dropped and dilated | Ulcer excised. Path. report non-malignant | Writes, 4.1911: "Very much better. I occasionally get bad attacks of indigestion." Can usually take ordinary food. |

18

| | | | | | | |
|---|---|---|---|---|---|---|
| ,, | *M. B.* | *26* | *F.* | *Pain just after food 10 years, in hospitals. Vomited blood 3 times years ago. HCl deficient* | *Hard pyloric ulcer. No-loop gastro-jejunostomy* | Writes, 4.1911 : "Relief for a few months." Now gets pain and vomiting. Diets carefully. |
| ,, | T. E. | 62 | M. | Pain 30 years, has to rest after meals. Vomited blood 10 years ago. No vomiting. HCl present | Ulcer of post. wall, excised. Path. report simple | Writes, 4.1911 : "No better, often vomits, lives on soft food. Laparotomy in Bridgwater, no relief. |
| 1910 (June) | K. C. | 43 | F. | Has had nephrorrhaphy. Pain 4 months, vomited everything since admission. Dilated dropped stomach | Pyloric ulcer. Antiperistaltic, no-loop gastro-jejunostomy | Writes, 4.1911 : "Vomiting returned after a fortnight. Not much pain." |
| 1910 (Mar.) | J. H. | 36 | M. | Pain 2 hours after food 12 years. Vomits daily. One large hæmatemesis. Dilated stomach. Lavage 2 years | Pyloric ulcer, nearly perforated. Antiperistaltic, no-loop gastro-jejunostomy | Writes, 4.1911 : "Real relief." Is "in splendid health." Takes ordinary food, no symptoms. |
| 1910 (June) | H. W. | 50 | F. | Old dyspeptic. Severe pain and frequent vomiting 1 year. Wasting. Lives on slops | Pyloric ulcer. Antiperistaltic, no-loop gastro-jejunostomy | Writes, 4.1911 : Is quite cured, and eats anything. |
| 1910 (April) | E. H. | 49 | F. | Pain, vomiting, wasting 6 months. Twice vomited blood. Melæna now. HCl present | Ulcer in lesser curvature. Isoperistaltic, no-loop gastro-jejunostomy | Writes, 4.1911 : Complete success. Takes ordinary food, and works well. |
| 1910 (Feb.) | E. S. | 27 | M. | From Medical ward. Pain 2 years ; three attacks of hæmatemesis, last 7 weeks ago | Pyloric ulcer. No-loop gastro-jejunostomy | Writes, 4.1911 : "Real relief." One attack of slight indigestion since, now well. Edentulous. |
| 1910 (Dec.) | A. B. | 35 | F. | Violent pain 1 hour after food, vomiting frequently. Dilated stomach | Ulcer of ant. wall. No-loop gastro-jejunostomy | Seen 5.1911. Quite free from symptoms, takes ordinary food. Operation "most successful." |
| 1910 (Oct.) | M. B. | 41 | F. | Dyspepsia 2 years, vomiting daily, wasting. Living on slops. Lump felt. Enormous stomach | Two hard ulcers, one in pylorus, one in lesser curvature. ? Cancer. Isoperistaltic, no-loop gastro-jejunostomy | Writes, 4.1911 : "Cured, no pain or vomiting, can eat anything. Getting fat." |
| 1910 (Nov.) | E. G. | 39 | F. | From Medical ward. Hæmatemesis, 1 quart. Pain after food 1 year | Pyloric ulcer. No-loop gastro-jejunostomy | Writes, 4.1911 : "A little pain sometimes, but I have not found anything of the old trouble since. |
| 1910 (Jan.) | N. J. | 29 | F. | Pain 1 hour after food, relieved by food. No vomiting. Pain has been very severe. Mass near umbilicus (found to be adhesions). Free HCl present | Pyloric ulcer. Gastro-duodenostomy | Lost sight of. |

SYMPTOMS OF GASTRIC ULCER: NO ULCER FOUND.

| Year. | Initials. | Age. | Sex. | Symptoms. | Findings and Treatment. | End-Results. |
|---|---|---|---|---|---|---|
| 1903 | J. G. | 54 | M. | From Medical ward. Continual copious vomits. Losing weight. Lavage useless | Dilated stomach. Gastro-jejunostomy | " Better than for years," when he left. Lost sight of. |
| 1904 | S. P. | 30 | F. | Recurring attacks of pain and vomiting 6 years; vomited much blood 2 months ago. Pain just after food. In several hospitals. Vomiting becoming incessant. HCl 0.03 % | Isoperistaltic, gastro-jejunostomy with loop | Great relief until 1909, when she came in again for vomiting. Band of adhesions divided. Anastomosis admitted 3 fingers. Writes, 4.1911: " The operation did me a lot of good." Gets pain and sickness if she takes meat or vegetables, not otherwise. |
| ,, | J. S. | 27 | F. | Pain 10 minutes after food. Vomited blood 6 times. Ill 8 years. No solids for months. | Isoperistaltic, gastro-jejunostomy with loop | Little relief for 6 months, then all symptoms returned. Had appendix removed in Bath. Is now, 4.1911, rather better, does not vomit. Gets some pain. Cannot work much, chronic invalid. |
| 1906 | R. W. | 30 | F. | Dyspepsia 12 years. 5 years ago in bed a month for severe vomiting. Frequent vomiting since, 2 hours after food. Some pain | Pylorus ? a little thickened. Gastro-jejunostomy | Relieved at the time. Lost sight of. |
| ,, | C. G. | 37 | F. | Pain soon after food and vomiting for 16 years. Vomited blood 8 years ago. Vomiting 10 times in a day, severe pain | Gastro-duodenostomy .. .. .. | Writes, 4.1911: " Did me much good for some months " (about nine). Now gets about 2 attacks a year of severe pain. " Most careful as to diet." |

| | | | | | | |
|---|---|---|---|---|---|---|
| ,, | F. A. | 45 | F. | Pain for 6 years, prolonged rest in bed several times. No vomiting. Movable kidney | Some gastroptosis. Finney's operation, gall-bladder explored | After 24 hours was given peptonised milk. Began to vomit blood, which went on for 16 hours till she died. *Nil* at *post-mortem.* |
| ,, | A. H. | 32 | M. | Was in Medical ward 4 years ago for pain, diarrhœa, and vomiting blood. Another attack a year ago. Now a third attack. Costive, pain, a little hæmatemesis | Stomach rather distended. Antiperistaltic, no-loop gastro-jejunostomy | Well for 9 months, then pain and vomiting recurred. Has had syphilitic rupia and laryngitis since. Seen 4.1911. Still some trouble, but considers operation relieved him. |
| 1907 | A. S. | 37 | F. | Hæmatemesis 9 months ago, in bed 6 weeks. Now dyspepsia and vomiting. HCl deficient, lactic acid present | Gastro-duodenostomy . . . . | Writes, 4.1911 : “ Cured, has better health now than ever in her life. No pain or vomiting. Avoids meat and vegetables. |
| ,, | E. G. | 42 | M. | Treated as gastric ulcer at General Hospital and in Medical ward for recurrent attacks of vomiting and hæmatemesis. Ill 6 years | Antiperistaltic, no-loop gastro-jejunostomy | Not improved. Was in Medical ward in 1909 for gastric crises of tabes. Now in imbecile ward at Eastville Workhouse. |
| 1907 | J. M. | 32 | M. | Pain and vomiting 1 year. Hyperchlorhydria | Antiperistaltic, no-loop gastro-jejunostomy | Writes, 4.1911 : “ The operation made a new man of me.” No symptoms since. “ Can eat anything and enjoy it.” |
| 1908 | K. F. | 28 | F. | Vomited blood in 1904. Vomiting and hunger-pain for 2 years. Weight 6 st. 12 lb. | ? Scarring of pylorus. Antiperistaltic, no-loop gastro-jejunostomy | At first better. Writes, 4.1911 ? Vomits every fortnight, once a little blood. No pain. Diet very restricted. Weight, 7 st. |
| 1910 (Dec.) | E. T. | 34 | F. | Attacks of pain and vomiting. On low diet, but still has pain. Dilated dropped stomach | ? Thickened pylorus. Antiperistaltic, no-loop gastro-jejunostomy | Writes, 4.1911 : No pain or vomiting, better. Still some “ indigestion after food.” “ Keeps catching cold.” |

| Year. | Initials. | Age. | Sex. | Symptoms. | Treatment. | End-Results. |
|---|---|---|---|---|---|---|
| 1905 | A. R. | 45 | M. | Dyspepsia many years. A year ago, and again 4 months ago, prolonged attacks of vomiting and hæmatemesis. Continues to vomit on nutrients | Isoperistaltic gastro-jejunostomy, with loop | Died on 6th day unexpectedly. A little pericarditis and perihepatitis ; œdema of the lungs at *Post-mortem*. |
| 1908 | C. W. | 52 | F. | In Medical ward for hæmatemesis. Pain, losing weight. Melæna. HCl normal | Antiperistaltic, no-loop gastro-jejunostomy | Writes, 4.1911 : Quite well, can take anything. No symptoms at all. |
| ,, | J. W. | 46 | M. | Dyspepsia and vomiting, probably melæna, for 6 months. HCl normal | Gastro-duodenostomy .. .. .. | Relieved at the time. Lost sight of. |
| 1910 (Mar.) | W. T. | 27 | M. | Pain 2 hours after food for 6 years, relieved by food. Lately vomiting frequently. HCl excessive. On fluids | Antiperistaltic, no-loop gastro-jejunostomy | Writes, 4.1911 : " A splendid cure." Can eat anything. Weighs 11 st. 6 lb. |
| 1910 (Feb.) | J. N. | 38 | M. | Pain and vomiting for 2 years, at first in attacks, now constantly. Relieved by food. Losing weight. Free HCl abundant. No occult blood in stools | Antiperistaltic, no-loop gastro-jejunostomy | Writes, 4.1911 : " Operation has given permanent relief." No pain or vomiting except after stooping or digging. Avoids cooked vegetables. |
| 1910 (June) | W. F. | 45 | M. | Very severe pain and vomiting 13 months, no relation to food. Once hæmatemesis. Often requires morphia. Explored a year ago, adhesions divided, no better since | Isoperistaltic, no-loop gastro-jejunostomy | Writes, 4.1911 : " Has really cured me. Not the slightest pain or vomiting since." Takes ordinary food. Has a cough ; was suspected of phthisis. |

| Year. | Initials. | Age. | Sex. | Symptoms. | Treatment. | End-Results. |
|---|---|---|---|---|---|---|
| 1907 | A. H. | 35 | F. | Pain for many years, worse lately. takes only fluids. Vomited blood 3 years ago. Was in General Hospital for gastric ulcer | Duodenal ulcer and hour-glass stomach. Gastroplasty; gastro-jejunostomy of proximal pouch | Writes, 4.1911: Greatly improved, "very thankful she had the operation done." Still some pain, has to diet carefully. "Real relief." |
| 1908 | S. B. | 40 | F. | Pain 15 years, vomiting. Free HCl present. Stomach dilated | Gastro-jejunostomy of proximal pouch | Died 8 days after of lung complications. |
| ,, | G. B. | 35 | M. | Pain and vomiting many years, pain just after food. Lavage 6 years. Stomach large | Pyloric obstruction, also slightly hour-glass. Gastro-jejunostomy | For 2 years no better; pain and vomiting are now better. Weight up and down. Diets carefully. |
| 1910 | R. H. | 35 | F. | Always dyspeptic. Vomited blood 5 months ago, in bed ever since with pain and daily vomiting; some blood. Melæna. Has Graves's disease | Extreme hour-glass contraction, large ulcer. Gastro-jejunostomy of proximal pouch | Ulcer perforated some weeks later; operated on, but died. Had been vomiting throughout. *Post-mortem*, phthisis. |
| 1910 (June) | S. W. | 42 | F. | Always dyspeptic. Vomited blood 21 years ago, and three severe attacks since. Pain for 2 months. No better with bed and fluids | Hour-glass contraction, with ulcer. Gastroplasty, distal gastro-jejunostomy | Writes, 4.1911: Quite cured, eats anything. Only complains of pricking sensation in the scar. |
| 1910 (Oct.) | N. W. | 35 | F. | Pain, frequent small vomits streaked with blood, not improved in Medical ward by massage, etc. 10 years' symptoms. Diagnosed as hour-glass by X-rays after bismuth | Marked hour-glass contraction. Double gastro-jejunostomy | Writes, 4.1911: Says she is "another woman, and getting fat." No pain or vomiting. avoids meat. Once or twice a little vomiting. |
| 1910 (Oct.) | E S. | 22 | F. | Always dyspeptic, twice in hospital. Pain 1 hour after food, frequent vomiting. Once hæmatemesis, 1 pint | Gastro-jejunostomy of proximal pouch | Writes, 5.1911: Quite cured, no symptoms at all. |

| Year. | Initials. | Age. | Sex. | Symptoms. | Findings and Treatment. | End-Results. |
|---|---|---|---|---|---|---|
| 1907 | B. T. | 24 | F. | After trifling dyspepsia, three severe attacks of hæmatemesis, in spite of bed and only rectal feeding. Desperately ill | Nothing abnormal found. Gastro-jejunostomy | After a severe illness recovered completely. Is now quite free from symptoms. No trouble since operation. |
| ,, | J. N. | 68 | M. | Vomited blood 3 years ago, and again now. Red blood corpuscles 2,760,000. Losing flesh. No pain | Nothing abnormal except adhesions. Gastro-jejunostomy | Writes, 4.1911: Perfectly well, no symptoms. "Can walk five miles." |
| 1910 (Jan.) | G. K. | 59 | M. | Severe hæmatemesis and melæna 22 days after operation for perforated ulcer. Old dyspeptic | Gastro-jejunostomy 3 weeks later | Got bronchitis. Writes, 4.1911: Perfectly well ever since. Eats anything. |
| 1910 (Feb.) | F. F. | 23 | F. | Four previous attacks of hæmatemesis, last in 1908. Was in-patient. Now some pain, followed by repeated vomiting of blood. One large hemorrhage | Nothing abnormal found. Gastro-jejunostomy | Lost sight of. |
| 1910 (May) | D. W. | 38 | F. | Pain in 2 hours after food, many years; vomiting. In Medical ward for three attacks of hæmatemesis, continuing on nutrients | Nothing abnormal found. Gastro-jejunostomy | Writes, 4.1911: "Operation in every way most successful." Takes any food with no trouble. |
| 1910 (Oct.) | A. A. | 32 | F. | Pain and a little vomiting 6 months. Was in General Hospital on Lenharz's diet for hæmatemesis. Admitted to Medical ward for another attack. 1½ pints vomited, and more next day | Pylorus rather thickened, ? ulcer. Gastro-duodenostomy | Writes, 4.1911: Relieved as far as stomach symptoms are concerned, but has a painful sinus. |
| 1910 (Nov.) | M. S. | 22 | F. | Always dyspeptic, severe pain and vomiting 4 weeks. Twice vomited blood in Medical ward | Pyloric ulcer. Antiperistaltic, no-loop gastro-jejunostomy | Seen 5.1911. Some nausea. feeling of tingling in scar after food. No pain or vomiting. |

### PERIGASTRIC ADHESIONS.

| Year. | Initials. | Age. | Sex. | Symptoms. | Findings and Treatment. | End-Results. |
|---|---|---|---|---|---|---|
| 1907 | M. V. | ? | F. | Was in for pain and vomiting 14 years ago ; suffered ever since. Lump and visible peristalsis. From Medical ward | Adhesions. Large stomach. No ulcer. Gastro-jejunostomy | Relieved. Since lost sight of. |
| 1910 | J. B. | 44 | M. | Pain just after food, and vomiting 1 year. Once vomited blood. Wasting. Dilated stomach | Large stomach adherent to gall-bladder. Gastro-jejunostomy | Relieved, sent to Convalescent Home. Sent back dying of bronchiectasis. |

### CONGENITAL STENOSIS OF PYLORUS.

| Year. | Initials. | Age. | Sex. | Symptoms. | Findings and Treatment. | End-Results. |
|---|---|---|---|---|---|---|
| 1907 | C. S. | 6 weeks | M. | Breast-fed. Constipation, forcible vomiting, visible gastric peristalsis. Pylorus felt once. Treated by peptonised milk, lavage, and Tr. Opii, but got worse. Lost 1 lb. in fortnight | Very thickened pylorus. Pyloroplasty | Did well, put on six ounces in 3 weeks, then ceased to improve, and died from no obvious cause. |

